# Hyperglycemia-Induced Inhibition of DJ-1 Expression Compromised the Effectiveness of Ischemic Postconditioning Cardioprotection in Rats

**DOI:** 10.1155/2013/564902

**Published:** 2013-11-04

**Authors:** Min Liu, Bin Zhou, Zhong-Yuan Xia, Bo Zhao, Shao-Qing Lei, Qing-Jun Yang, Rui Xue, Yan Leng, Jin-Jin Xu, Zhengyuan Xia

**Affiliations:** ^1^Department of Anesthesiology, Renmin Hospital of Wuhan University, 99 Zi Yang Road, Wuhan, Hubei 430060, China; ^2^Department of Anesthesiology, The First Affiliated Hospital of University of South China, 69 Chuan Shan Road, Hengyang, Hunan 421001, China; ^3^Department of Cardiac Surgery, Chongqing Zhongshan Hospital, 312 Zhongshan Road, Chongqing 400013, China; ^4^Department of Anesthesiology, The University of Hong Kong, Hong Kong; ^5^Department of Anesthesiology, Affiliated Hospital of Guangdong Medical College, Zhanjiang, Guangdong 524001, China

## Abstract

Ischemia postconditioning (IpostC) is an effective way to alleviate ischemia and reperfusion injury; however, the protective effects seem to be impaired in candidates with diabetes mellitus. To gain deep insight into this phenomenon, we explored the role of DJ-1, a novel oncogene, that may exhibit powerful antioxidant capacity in postconditioning cardioprotection in a rat model of myocardial ischemia reperfusion injury. Compared with normal group, cardiac DJ-1 was downregulated in diabetes. Larger postischemic infarct size as well as exaggeration of oxidative stress was observed, while IpostC reversed the above changes in normal but not in diabetic rats. DJ-1 was increased after ischemia and postconditioning contributed to a further elevation; however, no alteration of DJ-1 was documented in all subgroups of diabetic rats. Alteration of the cardioprotective PI3K/Akt signaling proteins may be responsible for the ineffectiveness of postconditioning in diabetes. There is a positive correlation relationship between p-Akt and DJ-1 but a negative correlation between infarct size and DJ-1, which may partially explain the interaction of DJ-1 and IpostC cardioprotection. Our result indicates a beneficial role of DJ-1 in myocardial ischemia reperfusion. Downregulation of cardiac DJ-1 may be responsible for the compromised diabetic heart responsiveness to IpostC cardioprotection.

## 1. Introduction

Ischemic heart disease, one of the major cardiovascular complications, is a leading cause of mortality in diabetic disease. Large evidence showed oxidative stress induced by hyperglycemia was the major mechanism contributing to the development and progression of myocardial infarction in diabetes mellitus (DM) [1]. Reperfusion therapies (coronary artery by-pass grafting, angioplasty, stent placement, or thrombolysis) when applied expeditiously restore coronary flow and limit cardiac dysfunction and infarct size. However, despite this, reperfusion also elicits pathophysiological changes responsible for more tissue injury after restoration of blood flow due to further aggravated oxidative damage [2]. The underlying mechanisms by which oxidative stress exerts adverse effects in myocardial ischemia reperfusion remain incompletely understood. Ischemic postconditioning provides protective effect against ischemia/reperfusion injuries, which is associated with a reduction in reactive oxygen species (ROS) generation, lipid peroxidation, and intracellular and mitochondrial Ca^2+^ overload [3]. Compared with ischemic preconditioning, postconditioning is a more promising approach to cardioprotection due to the difficulty to predict the onset of myocardial ischemia in clinical practice. Clinical data strongly supports an increased susceptibility to myocardial ischemia-reperfusion injury in patients with diabetes mellitus [4]. The risk of postmyocardial infarction death is increased 2- to 4-fold in diabetic patients compared to those without diabetes [5, 6]. However, postconditioning seems to lose its cardioprotective effect in subjects with diabetes, while the underlying mechanism is largely unknown. 

DJ-1, which was initially discovered as a novel oncogene and first reported in 1997 [7], extensively exists in most rodent and human tissues, such as brain, heart, kidney, liver, pancreas, and skeletal muscle [7]. Early studies about DJ-1 also revealed a main dwelling in familial Parkinson' disease. Gratifying, several latest research contributed to a progression in understanding the role of DJ-1 in oxidation and antioxidation. Recently, Jeong et al. [8] demonstrated by immunohistochemical analysis that transduced cell permeable Tat-DJ-1 fusion proteins prevented neuronal cell death in ischemic brain injury. Moreover, Yu et al. [9] found that stable overexpression of DJ-1 attenuated ischemia/reperfusion-induced oxidative stress in H9C2 cells under a hypoxia condition. All these studies suggested that DJ-1 has the antioxidative effect. However, whether or not DJ-1 expression was inhibited in diabetic heart remains unclear. In the present study, we hypothesized that reduction of DJ-1 expression aggravates ischemia reperfusion injury and attenuates the cardioprotective effects of postconditioning in diabetes.

## 2. Materials and Methods

### 2.1. Induction of Diabetes and Myocardial Ischemic Model

108 healthy adult male Sprague-Dawley rats (aged 12 weeks) of SPF level weighing between 250 ± 10 g were obtained from HUNAN SLAC JD Laboratory Animal Co. Ltd. All the animals were randomly divided into 8 groups: normal blank control group, diabetic blank control group, normal rats sham operated (NS group), normal rats subjected to MI/R (NIR group), ischemic postconditioning group (NIPO group), diabetic rats sham operated (DMS group), diabetic rats subjected to MI/R (DMIR group) and ischemic postconditioning group (DMIPO group). After equilibrated to surroundings for three days, diabetes was induced via single intraperitoneal injection of STZ (60 mg/kg, Sigma, St. Louis, MO, USA) dissolved in citrate buffer (0.1 M, pH 4.5), while the normal rats were injected equal volume citrate buffer alone. One week after STZ injection, rats exhibiting hyperglycemia (blood glucose ≥ 16.7 mM) were considered diabetic.

At the end of suffering DM for 12 weeks, the myocardial ischemia-reperfusion injury model was established by the left anterior descending (LAD) coronary artery occlusion. The sham groups (N + S group and DM + S group) that underwent isolation of the LAD without occlusion and the IR groups (both N + IR group and DM + IR group) subjected to 30 min myocardial ischemia and 2 h reperfusion after the LAD had been isolated and occluded while the IPO groups (both N + IPO group and DM + IPO group) were subjected to 3 cycles of 10 s occlusion/10 s reperfusion after myocardial ischemia.

### 2.2. Determination of Myocardial Infarct Area

The infarction area was measured by Evans blue and 2,3,5-Triphenyltetrazolium chloride (TTC) double staining: after the animals were sacrificed, the ligature around the coronary artery was reoccluded and 2 mL of 0.25% Evens Blue dye was injected into the aorta to map the normally perfused region of the heart. The myocardial area at risk (AAR) for infarction was delineated by the area of myocardium not dyed. The presence of Evans blue was used to identify the area that was not subjected to the ischemia. Rat hearts were rapidly excised and frozen at −20°C and then sliced into 2 mm thick sections perpendicular to the heart base-apex axis using a heart slice chamber. The slices were incubated for 15 min at 37°C in a phosphate-buffered 1% TTC (Sigma-Aldrich, St. Louis, MO) solution to determine infarcted myocardium. The viable tissue was stained red by TTC, while the infarct portion not taking up TTC stain remained pale. Morphometric measurements of the AAR and infarct area (IA) in each slice were performed with a scanner (Epson, v30, Japan) and an image analysis system (Image-Pro plus; Media Cybernetics, Bethesda, MA). The percentage of the infarcted area from the left ventricle (IA/LV) and the area at risk from the left ventricle (AAR/LV) were calculated.

### 2.3. Biochemical Assays

 Myocardial tissue was harvested and immediately homogenized on ice in 9 volumes of ice saline. The myocardial homogenate was centrifuged at 4,000 rpm for 10 min. The activity of Superoxide Dismutase (SOD) and the level of Malondialdehyde (MDA), 15-F_2t_-Isoprostane (15-F_2t_), and Total Antioxidant Capacity (T-AOC) in myocardial tissues were measured using SOD (Jiancheng, Nanjing, China), MDA (Jiancheng, Nanjing, China), 15-F_2t_-Isoprostane (Cayman Chemical Company, USA), and T-AOC (Jiancheng, Nanjing, China) assay kits according to the manufacturer's instructions.

### 2.4. Western Blot Analysis

Myocardium tissue samples (100 mg) were homogenized in lysis buffer with electric homogenate machine, and then the homogenates were centrifuged at 12,000 rpm for 15 mins at 4°C. After determining with BCA protein assay kit (Beyotime Biotech Inc., Jiangsu, China), the supernatants were used as protein samples. Samples containing equal amounts were separated on a 10% SDS-polyacrylamide gel, and then proteins were transferred to PVDF membrane. After blocked in 5% nonfat-dried milk for 1 h, membranes were incubated with rabbit anti-DJ-1 or anti-PTEN, anti-total Akt, and anti-phospho-Akt monoclonal antibody (1 : 1000 dilution, Cell Signaling Technology, USA) overnight at 4°C. Subsequently, the membranes were washed and incubated with the corresponding fluorescent tags goat anti-rabbit polyclonal IgG (1 : 10000 dilution, LI-COR, USA) for 1 h at room temperature. Open the Odyssey scanner and put the membranes facing down on the designated area for infrared fluorescence detection on the Odyssey Imaging Systems (LI-COR, USA). GADPH was chosen as a loading control to further assure the same volume for all the samples. 

### 2.5. Statistical Analysis

Mean ± SD values were calculated to summarize all outcome measurements. One-way analysis of variance (ANOVA) was used to compare significant differences among the groups, followed by Newman-Keuls Multiple Comparison Test. A value of *P* < 0.05 was considered to be statistically significant for all the statistical tests.

## 3. Results

### 3.1. General Characteristics of STZ-Induced Diabetic Rats

The general characteristics of diabetic compared with age-matched control rats are shown in Table 1. Before STZ induced diabetes, the body weight and fasting blood glucose (FBG) were recorded and no significant evidence was observed between the control and the intervention (*P* > 0.05, Table 1). 

During raising animal, we can observe that with a longer duration of diabetes, the diabetic rats were bald and showed polydipsia, polyuria, and weight loss. After feeding for 12 weeks, diabetic rats had remarkably lower body weight and higher blood glucose compared to the age-matched controls with euglycemia and a moderate growth rate at 5–10 g/d in normal group (*P* < 0.001, Table 1).

### 3.2. The Difference of Myocardial Infarcted Area between Normal and Diabetic Rats

**Figure 1:**
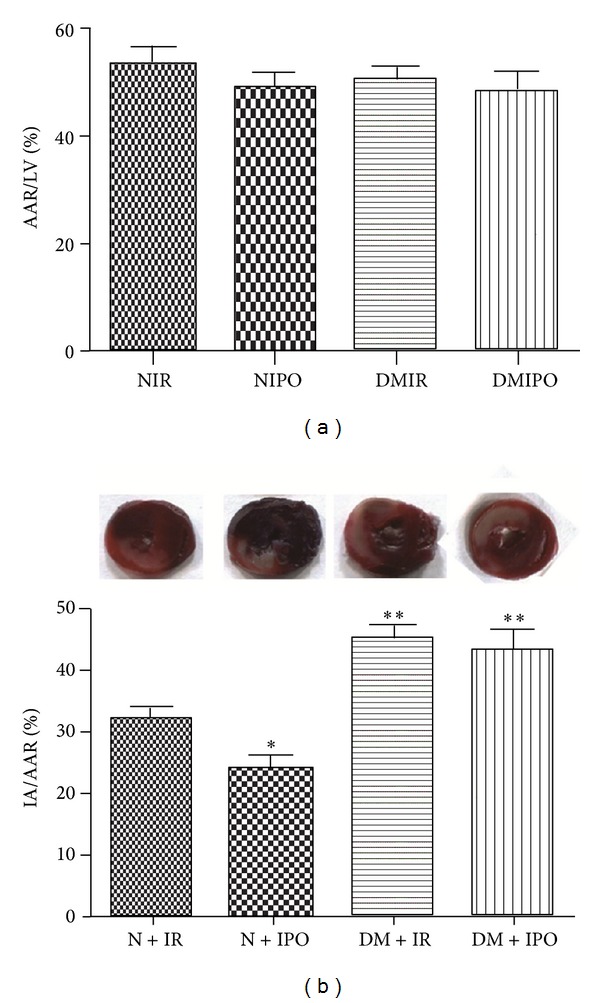
Shown are the area at risk from the left ventricle (AAR/LV) and infarcted area from the area at risk (IA/AAR) in percent means ± SD (*n* = 6 each). The blue-stained areas represent nonischemic tissue, and red stained areas represent the area at risk. Pale areas indicate infarct areas. (a) AAR/LV of normal and diabetic heart suffering from MI/R. There was no significant difference in AAR/LV between the four groups (*P* > 0.05). (b) IA/AAR of normal and diabetic heart which was subjected to MI/R with or without ischemic postconditioning. *P < 0.05 & **P < 0.01 versus N + IR group; no difference between DM + IR group and DM + IPO group.

To examine the different effect of cardioprotective postconditioning on the normal and diabetic hearts, myocardial infarcted area was measured. The representative images of AAR and IA from each group were shown in Figure 1. No more significant difference was shown in the size of area at risk (AAR/LV) among the four groups (Figure 1(a)). The infarct size in normal rats remarkably decreased with the protective effect of postconditioning (*P* < 0.05, Figure 1(b)). Interestingly, the diabetic heart shows more extensive infracted area percentage (IA/AAR) than normal heart when it suffers from the same degree of MI/R insult (*P* < 0.05, Figure 1(b)), and the cardioprotective effect of postconditioning was abolished since the infracted area percentage (IA/AAR) in DM + IPO group was similar to DM + IR group. These results provided that diabetic heart was more vulnerable to MI/R, and postconditioning cannot reverse the injury.

### 3.3. The Difference of Reactive Oxygen Species Level between Normal and Diabetic Rats

**Figure 2:**
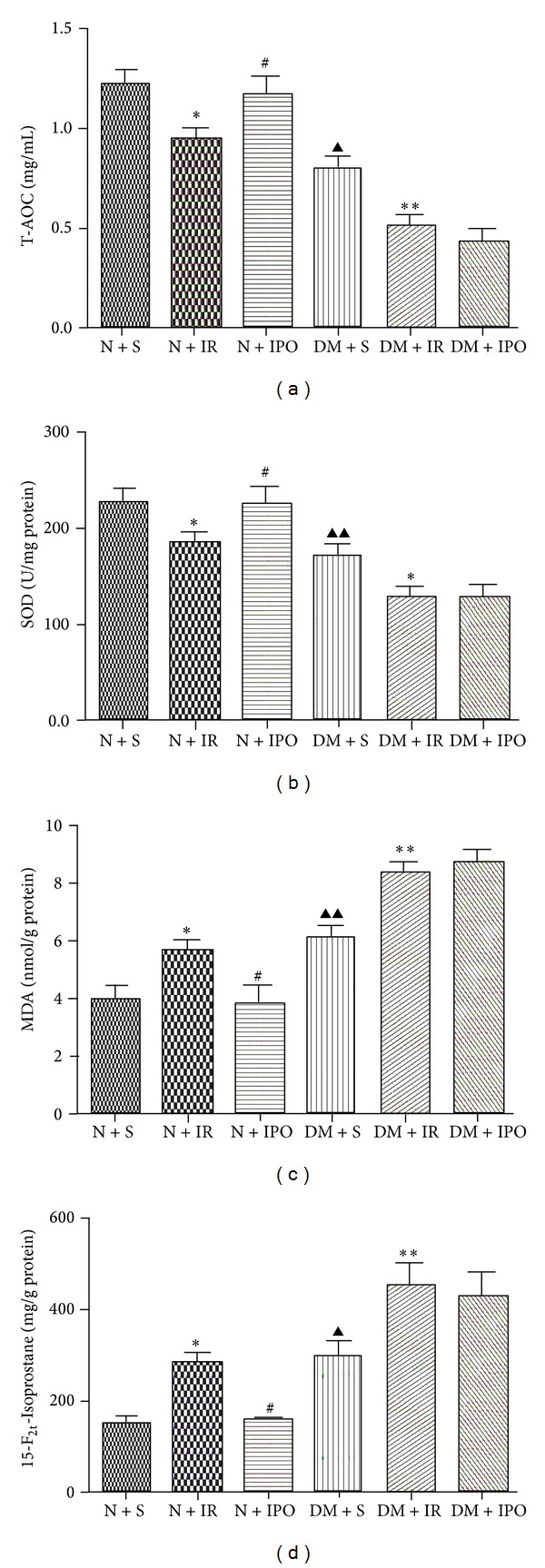
Index of oxidative stress in different groups was analyzed by ANOVA. (a) The level of Total Antioxidant Capacity (T-AOC). (b) The activity of Superoxide Dismutase (SOD). (c) The level of Malondialdehyde (MDA). (d) The level 15-F_2t_-Isoprostane. Values presented are mean ± SD (*n* = 6 each). ^▲^ *P* < 0.05 & ^▲▲^ *P* < 0.01 versus N + S group; *P < 0.05 & **P < 0.01 versus respective sham group^#^ *P* < 0.05 versus respective IR group.

Data are shown in Figure 2. Compared with the N + S group, the IR induced injury was manifested by a remarkable decrease in SOD and T-AOC, associate with an increase in MDA and 15-F_2t_-Isoprostane. The ischemic postconditioning caused a significant reversion of MI/R in normal rats. The diabetic rats got lower activity in SOD and the level of MDA, T-AOC and 15-F_2t_-Isoprostane in diabetic rats is significantly higher than normal rats. After subject to IR injury the activity of SOD losses mount in diabetic rats. Meanwhile the increasing level of MDA, T-AOC, and 15-F_2t_-Isoprostane in DM + IR group was aggravated. Unfortunately, ischemic postconditioning cannot restore the MI/R injury in diabetic rats.

### 3.4. The Difference of DJ-1 Expression between Normal and Diabetic Rats

**Figure 3:**
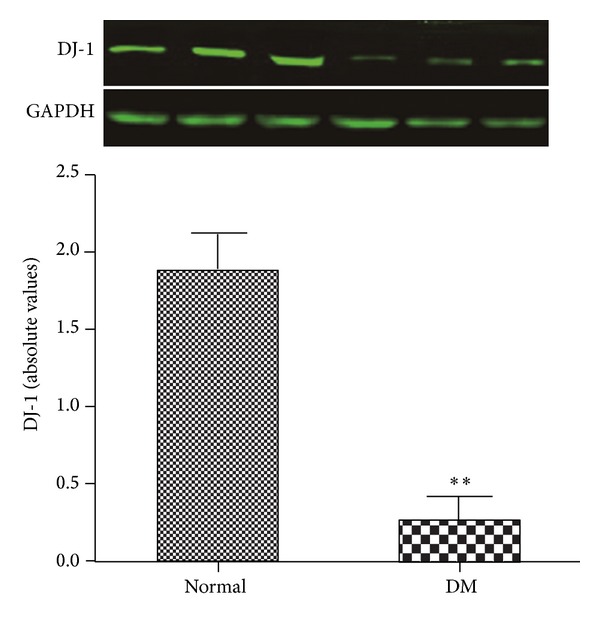
Effects of Diabetes Mellitus on the protein expression of DJ-1. There were three different normal blank control rats (on the left side) and three different diabetic blank control rats (on the right side). All values are expressed as mean ± SD (*n* = 6 each). **P < 0.01 versus normal rats.

It has been reported that DJ-1 is an antioxidative protein in ischemic brain injury [8]. To further investigate the different effect of postconditioning on normal and diabetic hearts, we attempted to determine the expression of DJ-1. As shown in Figure 3, the expression of DJ-1 was abolished by diabetes mellitus, when compared with the normal hearts and diabetic hearts, and this difference is not likely to occur by chance (*P* < 0.01). 

### 3.5. Variation of DJ-1 and Related Signal Transduction Molecules in PI3K/Akt Signal Pathway during MI/R and IPost in Normal and Diabetic Rats

**Figure 4:**
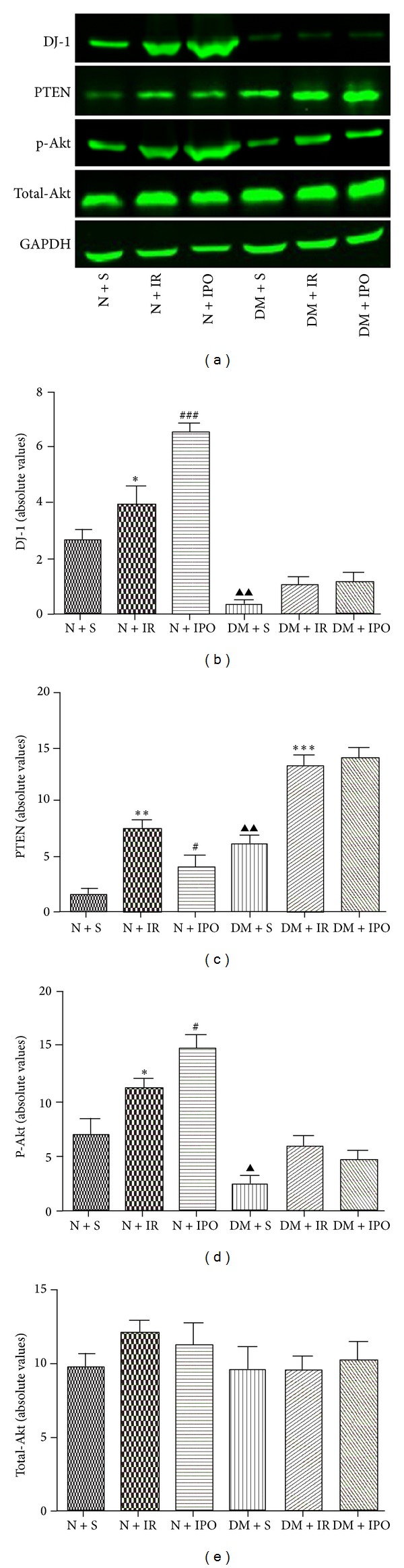
Immunoblot and densitometry analysis of the protein expression of DJ-1, PTEN, phosphorylated, and total Akt in normal and diabetic myocardial tissue. GAPDH was set as a loading control. Data are the mean ± SD (*n* = 6 each). (a) Western blot of DJ-1, PTEN, p-Akt, total Akt, and GAPDH. (b) Statistical analysis of DJ-1 among different groups. (c) Statistical analysis of PTEN among different groups. (d) Statistical analysis of p-Akt among different groups. (e) Statistical analysis of total Akt among different groups. ^▲^ *P* < 0.05 & ^▲▲^ *P* < 0.01 versus N + S group; *P < 0.05 & **P < 0.01 & ***P < 0.001 versus respective sham group; ^#^ *P* < 0.05 versus respective IR group; ^###^ *P* < 0.001 versus respective IR group.

Based on the recent studies in other system, DJ-1 may act as antioxidant agents associated with the regulation of PTEN and PI3K/Akt pathways [10], which are considered as important signaling systems involved in cardioprotection; thus, we investigated the expression of DJ-1 and related signal transduction molecules during MI/R and ischemic postconditioning in normal and diabetic rats. As shown in Figure 4(b), expression of DJ-1 in N + IR group was significantly higher than in N + S group (*P* < 0.01), and ischemic postconditioning further amplificated the increment between the N + IR group and N + IPO group (*P* < 0.001). However, when the same strike occurred in diabetic rats, no significant changes were identified in DJ-1 between DM + S group and DM + IR group (*P* > 0.05). In addition, the expression of DJ-1 was abolished by diabetes mellitus during ischemic postconditioning and no significant change was observed between DM + IR group and DM + IPO group (*P* > 0.05). The expression of PTEN was higher during MI/R (*P* < 0.01) and then inhibited when ischemic postconditioning aroused the level of DJ-1 in normal rats (*P* < 0.05). Furthermore, we also observed a high level of expression in MI/R in diabetic heart (*P* < 0.001) and drove the Akt activation at a significantly lower level (*P* < 0.05) due to the dysfunction of DJ-1, even ischemic postconditioning can never reverse (Figures 4(c) and 4(d)). As shown in Figure 4(e), no variation of total Akt was recorded in all groups (*P* > 0.05).

### 3.6. The Relationship between DJ-1 and Myocardial Infarcted Area, Phospho-Akt

**Figure 5:**
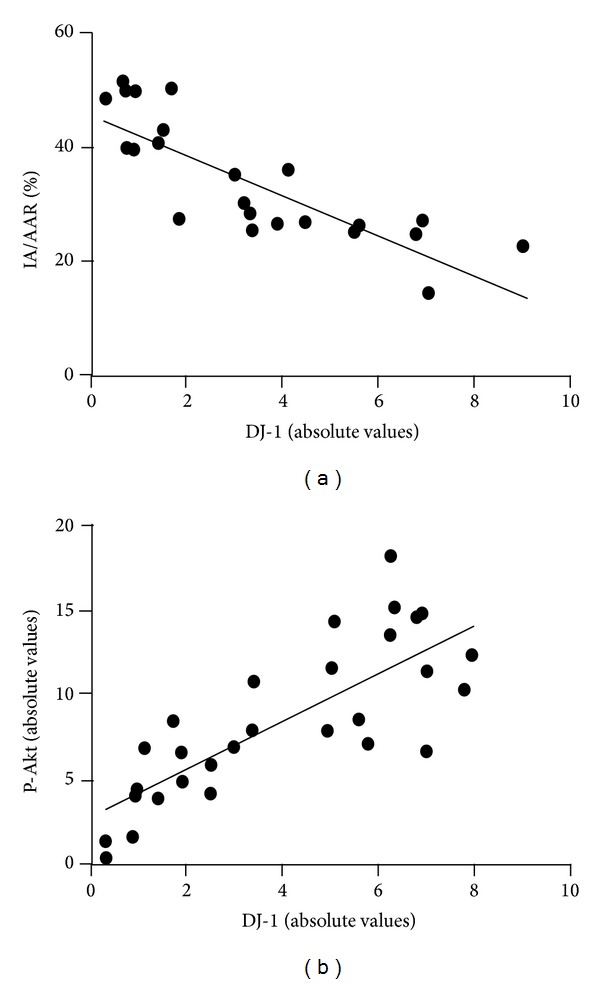
Simple linear regression between DJ-1 and myocardial infarct size together with p-Akt. (a) Linear regression between DJ-1 and IA/AAR. (b) Linear regression between DJ-1 and p-Akt.

Group differences in the relationship of DJ-1 expression and the myocardial injury indexes were evaluated with simple linear regression (Figure 5). The expression of DJ-1 was positively correlated with p-Akt with *r* ^2^ at 0.6331 (*P* < 0.001), while myocardial infarct size was negatively correlated with DJ-1; the corresponding *r* ^2^ was 0.6354 (*P* < 0.001). These results suggest that DJ-1 may be endowed with a salutary role in cardioprotection. 

## 4. Discussion

 We present several important observations from our study supporting the beneficial function of DJ-1 in myocardial defense against ROS. First, using *in vivo* models of MI/R, we demonstrated that the effectiveness of ischemic postconditioning as means of myocardial protection may closely related to the level of myocardial DJ-1. Second, to our knowledge, we are the first to report that the cardiac expression of DJ-1 was seriously inhibited in DM condition. Third, unlike the change of DJ-1 expression level in the normal rat, neither the compensatory increase of DJ-1 in response to the MI/R insult nor the protective increment in response to ischemic postconditioning was observed in diabetic condition.

In the present study, ischemic postconditioning reduced myocardial infarction size and oxidative stress level, which was intensely associated with enhanced expression of cardiac DJ-1. Our results are well in line with finding of other researches in kidney and brain [8, 11, 12]. Moreover, Lu et al. have recently demonstrated that hypoxic preconditioning upregulates DJ-1 protein expression in rat heart-derived H9C2 cells which contributes to the cardioprotection against hypoxia/reoxygenation injury [13]. Several lines of evidence suggest that overexpression of DJ-1 may have a crucial role against oxidative stress in ischemia/reperfusion (I/R) models. Regarding the increase in the expression of DJ-1 in normal MI/R group, we conjecture that it may be a compensatory increase during the MI/R injury.

It has been revealed that a reduction of antioxidative capacity in DJ-1 knock-out rats and mice *in vivo* and *in vitro* [14–16]. In our study we also exhibited a decline of T-AOC and SOD together with the increase of MDA and 15-F_2t_ in diabetic rats which may be due to the downregulation of DJ-1. Apart from the direct decreased expression of DJ-1, another possibility that may account for the exacerbation of oxidative stress was the peroxidation inactivation of DJ-1. Meulener and his colleagues [17] demonstrated that human DJ-1 (hDJ-1) rescues flies mutant for DJ-1b, and cysteine residue in the fly DJ-1 (C104) is analogous to C106 in hDJ-1. It has been confirmed in other studies that, the conserved cysteine residue in DJ-1 (Cys106) was both essential for DJ-1 protective function and was sensitive to oxidation [18]. It was supposed that the C104 subunit of DJ-1b was overoxidized in chemical toxin or aged condition, which resulted in the failure of dimerization and inactivation [17], similar to the one occurred in diabetic status in our study. Thus, the oxidative stress was further aggrandized due to the reduced activation of DJ-1 and consequently weakened antioxidative effect.

In the present study, the infarct size and oxidative stress were more extensive in diabetic rat hearts than in normal rats when endured the equivalent extent of ischemic reperfusion insult. The peroxidative inactivation of DJ-1 caused by excessive ROS will facilitate ROS overproduction in diabetes, which may predispose to a hypersensitivity of diabetic myocardium to ischemia and reperfusion in a vicious circle manner. 

Despite the emphasis on the impact of the duration of the diabetic state, metabolic disturbance, more extensive lesions (include atherosclerosis and hypertrophied), left ventricle dysfunction on myocardial injury in diabetes [19–22], and a number of studies which were carried out to investigate the myocardial protective signaling disrupted by diabetes have also been advocated [23, 24]. From data presented in the literature, two signal pathways have contributed to myocardial protection, one is Survivor Activating Factor Enhancement (SAFE) pathway and the other is Reperfusion Injury Salvage Kinase (RISK) pathway, which encompasses PI3K/AKT and ERK 1/2. Activation of PI3K/AKT pathway is responsible for protecting the myocardium by regulating essential cellular functions, such as cell migration, cell survival, and modulating several essential biologic processes, such as metabolism [25]. Furthermore, insulin activation of the PI3K/AKT pathway has been shown to delay the time to mitochondrial permeability transition pore (mPTP) opening and reduce ROS level [26]. Therefore, any strategy to activate PI3K/Akt and enlarge the threshold to mPTP opening may provide the protective effect against myocardial ischemia/reperfusion injuries. 

Despite that DJ-1 expression would be upregulated under myocardial ischemia and reperfusion injury, it was not enough to resist myocardial insult resulted from ischemia reperfusion, while postconditioning may add to an endogenous activation of the RISK pathway which enhances the antioxidative effects of DJ-1. However, chronic oxidation under DM may attenuate the expression of DJ-1 and the underlying RISK pathway may also be partially inactivated; thus, myocardial DJ-1 could not be stimulated under IR and subsequently the responsiveness to postconditioning could be attenuated or diminished.

Several studies have demonstrated that DJ-1 robustly protects cells from oxidative stress through distinct cellular pathways [27, 28]. In normal rats, ischemic postconditioning can arouse DJ-1 high expression and confer myocardial protection. Base on a series of studies on cancer cells, Kim et al. [10] found that DJ-1 functions in the PI3K/Akt (RISK) pathway as a negative regulator of PTEN. These results are in good agreement with ours. In the normal rats, ischemic postconditioning increased myocardial expression of DJ-1, paralleled with decreased expression of PTEN as well as increased Akt phosphorylation, and protected heart from MI/R, while in diabetic rats, DJ-1 expression was inhibited due to the overoxidation cause by DM. Therefore, ischemic postconditioning cannot reverse the DJ-1 expression in diabetic rats. As a result, the expression of PTEN highly grows unchecked and arrest PI3K/Akt (RISK) pathway functions. These fatal change lead to a reduction in the threshold and triggering mitochondrial permeability transition pore (mPTP) opening in response to Ca^2+^ overload and break the delicate balance between ROS production and scavenging capacity of antioxidant systems. Given the deficiency of agonist and antagonist of DJ-1, a better understanding of the interaction between DJ-1 and ischemia postconditioning protection could only be achieved by simple-linear regression; thus, further silencing and overexpression of DJ-1 via genetic engineering would be needed, which could add to a further comprehension of the underlying mechanism and may provide a promising alternative in the prevention and treatment of cardiovascular disease with DM.

## 5. Conclusion

With the result of *in vitro* study, we demonstrated the possible correlation between inhibition of DJ-1 induced by hyperglycemia and ineffectiveness of postconditioning in diabetic rats and that PI3K/Akt signal pathway may be one of the major factors involved in the protective effect of DJ-1, which should be further elucidated by *in vivo* studies. Our research revealed a potential curative role of DJ-1 in the treatment of diabetic cardiac complication. Thus, therapeutics aiming at upregulating DJ-1 could be considered as a novel alternative to alleviate the reperfusion injury in diabetic patients with ischemic heart disease.

## Figures and Tables

**Table 1:** Changes in body weight and fasting blood glucose  of control and diabetic rats before and after 12 weeks of STZ or vehicle injection.

	Normal		Diabetes	
	0 W	12 W	0 W	12 W
Body weight (g)	239.5 ± 7.4	418.7 ± 15.0	235.2 ± 6.7	204.5 ± 7.2***
FBG (mmol/L)	6.6 ± 1.1	7.5 ± 1.6	7.1 ± 1.2	25.7 ± 4.5***

All values are expressed as mean ± SD. ***P < 0.001 versus normal rats 12 weeks later after STZ injection.
